# Adherence to Antibiotic Prophylaxis for Percutaneous Transhepatic Cholangiography: A Single-Centre Experience

**DOI:** 10.7759/cureus.7989

**Published:** 2020-05-06

**Authors:** Debamita Bhattacharjee, Twishaa Sheth, Alfred Adiamah, Dhanwant Gomez

**Affiliations:** 1 Hepatobiliary Surgery, Queen's Medical Centre, Nottingham, GBR; 2 Hepatobiliary and Pancreatic Surgery, Queen's Medical Centre, Nottingham, GBR; 3 Hepato-Pancreato-Biliary Surgery, Nottingham University Hospitals, Nottingham, GBR

**Keywords:** antibacterial agents, prevention & control

## Abstract

Background

Percutaneous transhepatic cholangiography (PTC) is associated with high rates of sepsis. The aim of the current study was to audit the adherence to trust guidelines on antibiotic prophylaxis in patients undergoing PTC. Secondary aims included the management and outcome of patients with sepsis post-procedure.

Methods

This was a retrospective analysis of 50 consecutive patients who underwent a PTC procedure between January 2016 to January 2017. Collated data included PTC indication, drug allergies, antibiotics given pre-PTC, culture results, and antibiotic sensitivities within one-month post-PTC.

Results

Complete data were available for 41 patients and 61 PTC procedures. The median age was 68 years, and 51% were females. The indication for PTC was malignancy (n=32, 78%), benign conditions (n=1, 2%), and unknown diagnosis at the time of PTC (n=8, 20%). Three cases did not receive antibiotic prophylaxis. Only 27 (44%) PTC procedures received appropriate pre-PTC antibiotics as per guidelines, with no adherence to guidelines in all penicillin-allergic patients. In six patients who were not being treated for sepsis pre-PTC, a newly positive post-PTC blood/drain culture was observed within one month. Organisms grown in the post-PTC cultures were 56% gram-negative, the majority being Escherichia coli. The 30-day mortality rate was 12.2% (5/41).

Conclusions

Poor adherence to recommended antibiotic regimes is a significant contributing factor for sepsis post-PTC. Investigating barriers to guideline implementation, stricter adherence, and peer education are interventions that could improve post-PTC outcomes.

## Introduction

Percutaneous transhepatic cholangiography (PTC) involves the insertion of a needle through the liver into the biliary system and the injection of contrast to allow radiological visualisation of the biliary anatomy. This procedure can be used both for diagnostic and therapeutic purposes. In addition, this procedure is performed in cases where endoscopic retrograde cholangiopancreatography (ERCP) has failed or is contraindicated. Patients with altered anatomy following surgery that prevents endoscopic access to the biliary tree (for example, gastric bypass procedures, pancreaticoduodenectomy, and liver transplantation) are also candidates for PTC. Therapeutic indications include palliative biliary stenting across malignant strictures, dilatation of post-operative strictures, and removal of gallstones [1]. However, careful patient selection is required, as this procedure can be associated with significant complications.

Sepsis is an important, known complication following PTC and is a leading contributor to morbidity and mortality post-procedure. The current published literature suggests that post-PTC sepsis is usually associated with gram-negative bacteraemia, which is thought to be related to the instrumentation of an infected, obstructed biliary system [2]. Although some clinicians have suggested using antibiotic prophylaxis prior to the procedure to prevent this complication, there is currently a lack of consensus with respect to the type and duration of antibiotic prophylaxis that should be administered [3-4].

A previous study based at our institution demonstrated a sepsis rate of 2% within 30 days of PTC stent insertion [5]. Other studies have reported higher sepsis rates following this procedure [6]. The current guidelines at our local hospital trust suggest piperacillin-tazobactam as the first-line antibiotic prophylaxis [7]. In patients who are penicillin-allergic, a combination of cefuroxime or ciprofloxacin and metronidazole is recommended. The aim of the current study was to audit the adherence to the trust guidelines of antibiotic prophylaxis in patients undergoing PTC. Secondary aims included the management and outcome of patients with sepsis post-procedure.

## Materials and methods

Patients admitted to Queen’s Medical Center at Nottingham University Hospitals NHS Trust, Nottingham, United Kingdom, who underwent a PTC during the one-year period from January 2016 to January 2017 were identified from the hospital’s Interventional Radiology database Computerised Radiology Information System (CRIS) using the procedure code: FPTCH. Exclusion criteria included paediatric patients (less than 16 years old), incomplete PTC procedures, missing prescription data and patients undergoing any other surgical procedure.

This was a registered Clinical Audit (No: 17-039c) and the reference standard used was identified from the hospital’s intranet: “Nottingham University Hospitals NHS Trust. Clinical Guideline for Antibiotic Prophylaxis in Adult Gastrointestinal Endoscopy. December 2015” [7]. The guidelines recommend a treatment course of intravenous (IV) piperacillin-tazobactam 4.5 g three times a day for routine antibiotic prophylaxis in all patients undergoing PTC who are not allergic to penicillin. If the patient is allergic to penicillin, the biliary sepsis guidelines recommended the administration of intravenous cefuroxime 1.5 g three times a day or intravenous ciprofloxacin 400 mg BD with intravenous metronidazole 500 mg three times a day.

Collated data included patient demographics, ethnicity, co-morbidities, clinical presentation, waiting time between request and procedure, drug allergies, antibiotic therapy prior to PTC, identification of the organism in blood/drain culture within one-month post-PTC, and antibiotic sensitivities of positive blood/drain culture within one month of the PTC procedure and clinical outcome.

Statistical analysis

Data were analysed using STATA/SE 15 software (StataCorp LLC, College Station, TX). Continuous variables were expressed as means and standard deviation, while categorical variables were represented as percentages. We calculated the relative risk reduction of post-PTC sepsis between patients receiving the appropriate and incorrect PTC prophylaxis.

This project evaluated clinical service provision within the department and in line with local policy and hence did not require prospective ethical approval representing a quality improvement exercise.

Peer intervention survey

**Figure 1:**
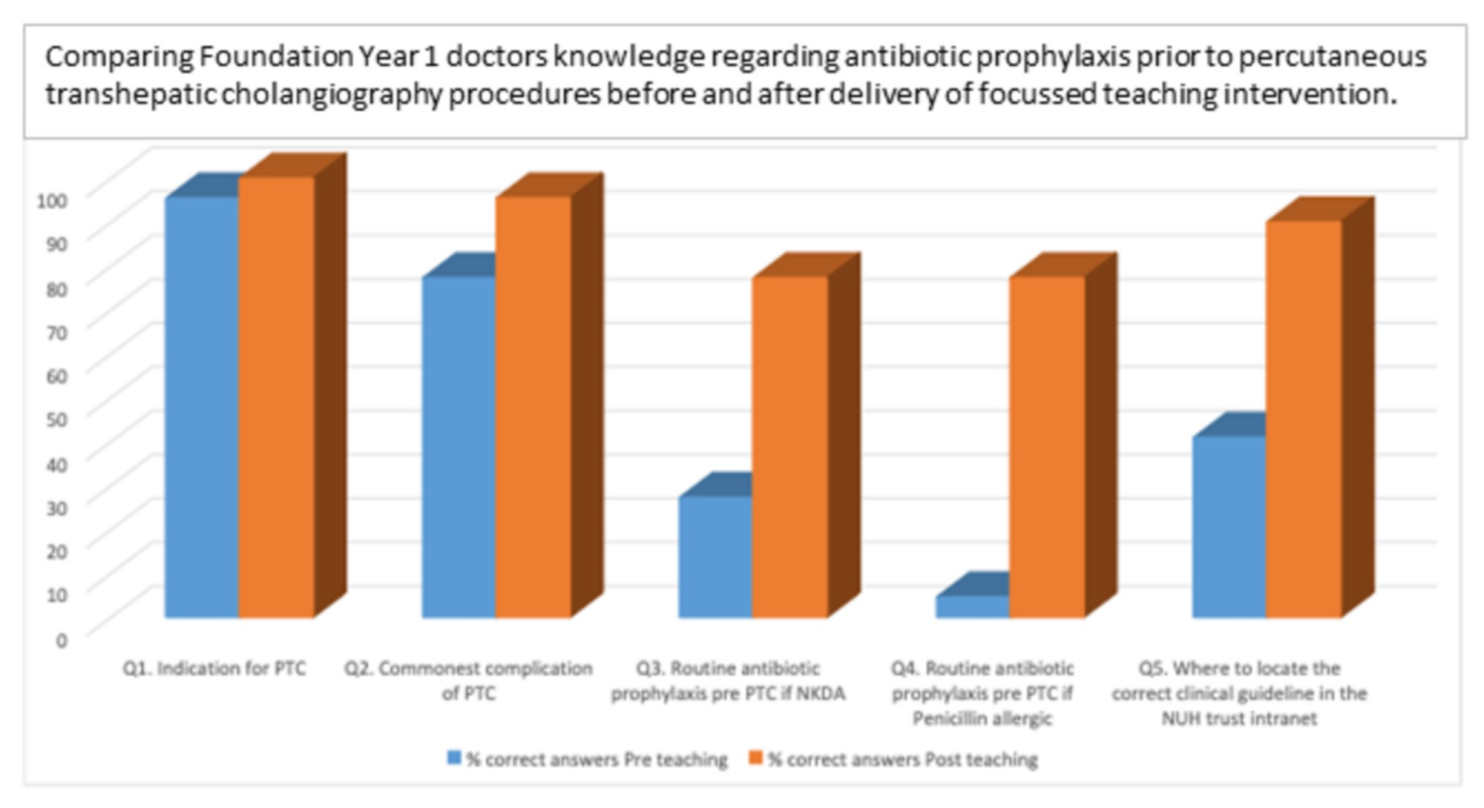
FY1 knowledge on antibiotic prophylaxis for PTC before and after delivery of peer education intervention FYI: foundation year 1; PTC: percutaneous transhepatic cholangiography

A peer education intervention was designed following the audit findings, with the aim to deliver focussed teaching to foundation year (FY) doctors on antibiotic prophylaxis for PTC. The target audience was asked to complete a pre-intervention survey assessing FY doctors’ knowledge of indications, complications, and knowledge of appropriate antibiotics in non-penicillin allergic and penicillin-allergic patients undergoing a PTC (Figure 1).

## Results

During the study period, 50 patients underwent PTC, of which 41 patients and 61 PTC procedures were included. Nine patients were excluded due to missing relevant drug charts (n = 6) and the procedure not performed (n = 3). The median age of patients included was 68 years (IQR: 61 - 70 year) and 21 (51%) patients were of the female gender. The majority of patients were Caucasians (n=30, 73%). The remaining patients were of black (n=1, 2.4%), Asian (n=1, 2.4%), and unknown ethnicity (n=9, 22%).

A total of 61 PTC procedures were performed in these 41 patients, and the indications included: underlying malignancy ((n = 32, 78%), pancreatic cancer (n=12, 37%), cholangiocarcinoma (n=11, 34%), metastatic carcinoma (n=5, 16%), upper gastrointestinal cancers (n=4, 13%)), benign conditions (n=1, 2%), and unknown aetiology at the time of PTC (n =8, 20%).

In the 61 PTC procedures performed, only in 27 (44%) cases did patients receive the appropriate pre-PTC antibiotic prophylaxis as recommended by the trust. Three (5%) of the PTC cases did not receive antibiotic prophylaxis. However, with respect to patients who were penicillin-allergic (n=5), none of these patients received the appropriate antibiotic therapy (Table 1).

**Table 1:** Basic demographics PTC: percutaneous transhepatic cholangiography

Parameters	Correct Prophylaxis	Incorrect Prophylaxis	Total (41 patients)
Demographic data			
Age, mean (SD), years			68 years
Gender			
Male, n (%)	10	10	20
Female, n (%)	10	11	21
Primary aetiology, n (%)			
Primary malignancy	10	16	26
Metastatic	5	1	6
Benign	0	1	1
Unknown	6	2	8
Medication allergy			
No allergies to penicillin	17	19	36/41
Penicillin allergy	0	5	5/41
Post PTC sepsis	4	3	7/41
30-day mortality	3	2	5/41

**Figure 2:**
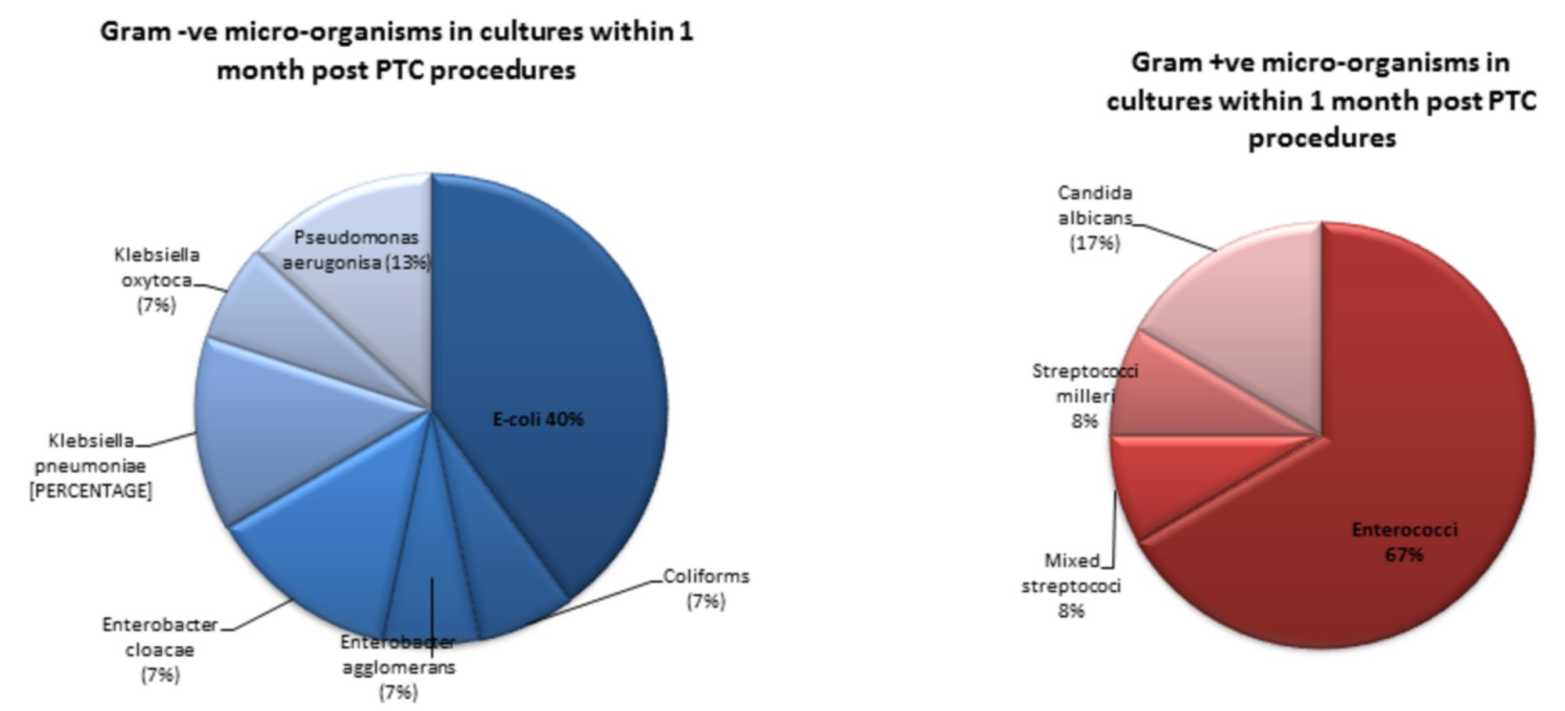
Pattern of microbiology growth in cultures within one-month post-PTC procedures 56% of all cultures grew gram-negative micro-organisms and 44% gram-positive. Within each group, the distribution of bacterial grown in percentages are presented. PTC: percutaneous transhepatic cholangiography

Six (22%) of the 27 patients who were not being treated for sepsis pre-PTC had a newly positive post-PTC blood/drain culture within one month. Organisms grown in the post-PTC cultures were 56% gram-negative (Escherichia coli commonest) followed by 44% gram-positive (Enterococci predominant, Figure 2).

There was a 72.7% relative risk reduction of developing post-PTC sepsis if patients were given the correct antibiotic prophylaxis pre-PTC compared to those patients that received no antibiotic prophylaxis. There was also a 50% relative risk reduction if patients were given antibiotic prophylaxis even if it was not the prophylaxis therapy recommended in the trust’s guidelines.

The median length of hospital stay following PTC prophylaxis was seven days (interquartile range 3-15 days). However, in those with post-PTC sepsis, the median length of stay (LOS) was 13 days (IQR 6.5 - 17 days). Overall 30-day mortality was 12.2% (5/41) and 16% (1/6) in those with post-PTC sepsis.

Peer intervention

There were low levels of FY1 knowledge (49.1% of correct answers) pre-intervention. Peer education improved FY1 knowledge on all points of knowledge assessed. Poorest pre-intervention (4.6%) was on routine pre-PTC antibiotic prophylaxis in penicillin-allergic patients, correlating with the audit, which showed this was the area of least compliance with trust guidelines when prescribing pre-PTC antibiotic prophylaxis. Of note, this was also the area of greatest improvement post-intervention (72.3% increase in correct answers, Figure 1).

## Discussion

Whilst PTC is an effective diagnostic and therapeutic intervention in the management of benign and malignant biliary tract disease, the associated morbidity and mortality remains high. Sepsis is one of the most important outcomes post-PTC, which further impacts overall morbidity, length of hospital stay, and mortality. 

In the present study, poor adherence with respect to the choice of antibiotic prophylaxis administered was observed, with only 44% adherence to the trust guidelines, and none of the penicillin-allergic group patients had appropriate antibiotic prophylaxis prescribed. In addition, there was a high rate of post-PTC sepsis based on positive blood or biliary cultures.

Published data have previously shown that the 30-day mortality for PTC ranges from 2%-19.8%, with post-PTC sepsis being a leading contributor to this poor outcome [8-11]. There may be multiple reasons for the development of post-PTC sepsis, including bacteraemia from the instrumentation of an infected obstructed system, as well as the vascular spread of bacteraemia due to the passage of the PTC needle. It has been postulated that when the normally sterile biliary system becomes obstructed and subsequently colonised with bacteria, usually gram-negative species, these bacteria can “intravasate” into the systemic bloodstream resulting in sepsis [12].

There are conflicting views on the effectiveness of antibiotic prophylaxis for interventional radiology procedures. The Society of Interventional Radiology recommends prophylactic antibiotics in all patients undergoing PTC and its respective biliary drainage procedures due to the anticipated rate of sepsis of 2% [13].

In contrast, American Society for Gastrointestinal Endoscopy does not advocate the use of prophylactic antibiotics for procedures intended to relieve biliary obstruction where there no signs of cholangitis pre-procedure and where complete drainage is expected [12].

There is also controversy in the literature as to the optimal antibiotic of choice to be used as prophylaxis for this purpose. Piperacillin-tazobactam has been proposed as a suitable prophylactic antibiotic due to its coverage of biliary bacterial flora, action against the B-lactamase producing species of Escherichia coli and low risk of nephrotoxicity [12]. This antibiotic is also our trust-recommended first-line antibiotic for pre-PTC prophylaxis, in patients with no penicillin allergy. However, third-generation cephalosporins have also been suggested as alternatives [3].

In the present study, there was poor adherence to the trust guidelines on antibiotic prophylaxis for PTC procedures. In addition, there was a higher rate of post-PTC sepsis compared to the older published literature [11]. However, when compared to more recent studies, the rate of post-PTC sepsis ranges from 26% to 46% [3,14]. The pattern of microbiology results found in our sample of patients is in line with previous studies, which have found a dominance of gram-negative bacteria, which should, in theory, be well-covered by the trust recommended choice of antibiotic for pre-PTC prophylaxis, which is currently piperacillin-tazobactam. The post-PTC microbiology in the blood and drain cultures found does not reflect postoperative infective complication as the patient population used for this study were those who were deemed unfit for surgery. Thus, the blood and drain cultures are likely to be a true reflection of the post-PTC infective complications that the patients experienced.

In the present study, there was a significant relative risk reduction in the development of post-PTC sepsis when patients were given the trust-recommended antibiotic prophylaxis when compared with those patients that received no prophylaxis. Of interest, there was also a significant relative risk reduction of post-PTC sepsis when incorrect antibiotic prophylaxis was given compared to when no antibiotic prophylaxis was administered.

The benefits of peer education as a model for delivering education has been supported by previous research and has been translated into the context of medical education [15]. Adult learning theories can be applied to explain the process of learning involved in near-peer education such as the one we describe in our study: With regards to the junior peer in this study, the presentation of a case on post-PTC sepsis by them added value to the peer-education intervention, as it provided context to the topic being taught, highlighted its importance particularly with reference to patient safety, emphasised its relevance (i.e. the critical role junior doctors can play in prescribing correct antibiotic prophylaxis) and hence played a role in motivating the audience and gaining their attention, the first step in Gagne’s nine events of instruction [16]. The peer education intervention improved the FY1 doctors’ knowledge of all aspects of knowledge assessed in the pre-intervention survey. Poorest adherence to trust guidelines was in patients allergic to penicillin, and this was also the subject of greatest improvement in FY1 doctors’ knowledge post-intervention.

Limitations

There are limitations to this study. This was a retrospective study, and the data collated represented a one-year period of practice in a tertiary centre, and hence the small sample size. It would, in the future, be important to re-evaluate using larger sample size, however, the findings of increased septic complications is of significant importance to this particular population of patients, 78% of whom had unresectable malignancy. 

Given that the study was conducted in a large tertiary referral centre that undertakes PTC procedures for the East Midlands region, it could be argued that results could be generalised to the wider UK population, however, the small sample size may limit this study's external validity. Randomising the sample selection using systematic sampling could limit selection bias. The exclusion of nine patients with incomplete data may have further introduced selection bias into our sampling.

A non-generalisable finding from this study is the type of antibiotic to be used for prophylaxis, as this is linked to local sensitivities and local antimicrobial policies and guidance.

## Conclusions

Poor adherence to recommended antibiotic prophylaxis regimes leads to higher rates of sepsis post-PTC with the sequelae of increased morbidity and length of stay. Therefore, investigating barriers to guideline implementation, stricter adherence and peer education are all interventions we anticipate could improve post-PTC outcomes.
